# Deliberating Upon the Living Wage to Alleviate In-Work Poverty: A Rhetorical Inquiry Into Key Stakeholder Accounts

**DOI:** 10.3389/fpsyg.2022.810870

**Published:** 2022-06-03

**Authors:** Darrin J. Hodgetts, Amanda Maria Young-Hauser, Jim Arrowsmith, Jane Parker, Stuart Colin Carr, Jarrod Haar, Siautu Alefaio

**Affiliations:** ^1^School of Psychology, Massey University, Albany, New Zealand; ^2^School of Management, Massey University, Albany, New Zealand; ^3^Department of Management, Auckland University of Technology, Auckland, New Zealand

**Keywords:** living wage, minimum wage, rhetoric, public deliberation, in-work poverty, socio-economic inclusion

## Abstract

Most developed nations have a statutory minimum wage set at levels insufficient to alleviate poverty. Increased calls for a living wage have generated considerable public controversy. This article draws on 25 interviews and four focus groups with employers, low-pay industry representatives, representatives of chambers of commerce, pay consultants, and unions. The core focus is on how participants use prominent narrative tropes for the living wage and against the living wage to argue their respective perspectives. We also document how both affirmative and negative tropes are often combined by participants to craft their own rhetorical positions on the issue.

## Introduction

Recognizing foundational links between work, income and poverty, over the past century most OECD countries have introduced statutory minimum wages (MW) as a key pillar of their social safety nets. These MWs were originally envisioned as living wages (LWs) that could lift people out of poverty and sustain the health, morale, and productivity of communities, and reduce state dependency (Levin-Waldman, 2000). Efforts to ensure such decent pay have sparked considerable public controversy. Powerful conservative interests have opposed livable wages by arguing that these are not financially viable for businesses, are not warranted due to low employee productivity, and will result in job losses and business failures (Fourcade, 2009; Karjanen, 2010; Skilling and Tregidga, 2019). Correspondingly, the value of the MW in many OECD countries has been eroded and kept artificially lower than the LW. This has resulted in increased in-work poverty (Schulten and Luebker, 2019). In response, LW advocates have mobilized research evidence that shows that higher wages need not lead to increased unemployment or business closures (Card and Krueger, 1995; Luce, 2004). In promoting decent pay as a solution to in-work poverty, LW campaigners have also mobilized broader social justice notions of business ethics, social license, equity, socio-economic inclusion, and human dignity (Luce, 2004; Stabile, 2008). These campaigns have gained considerable momentum with the 2008 global financial crisis and subsequent recession in many that was exacerbated by poverty generating austerity responses in many countries. The current COVID-19 pandemic has also highlighted the importance of “essential” low-paid workers and the in-work poverty they experience (Hopner et al., 2021).

Since antiquity, scholars have considered links between public understandings of such contentious issues and argumentation (Levine and Saunders, 1993). More recently, Wood (1997) explored key arguments for and against the introduction of the MW in the United Kingdom, as evident in media items and published research. Similarly, Karjanen (2010) explored LW debates in selected jurisdictions in the United States and London. Skilling and Tregidga (2019) found that the majority of relevant public reports, press releases and subsequent news items in New Zealand presented arguments for the LW, reflecting growing pressures from unlivable wages and high living costs (Plum et al., 2019). Arguably, this reflects an asymmetrical conflict whereby those seeking change have to advocate more vigorously to challenge this *status quo* of poverty level wages. Relatedly, those opposing the LW often simply defend the *status quo* by reducing the dialog, responding primarily to particular points of argument, and/or diverting attention elsewhere.

Research cited above has begun to explore rhetorical trends in wage setting in publicly available documents. We seek to extend such research by drawing on face-to-face conversations with key representatives (rhetors) of employers and employees. This article explores how these rhetors (Ancient Greek term for “orator,” Walker, 2000) argue for and against the LW in Aotearoa/NZ; one of the first countries to introduce a statutory MW that is now amongst the highest in the world (Schulten and Luebker, 2019). Conversely, our national average wage is relatively low (Organisation for Economic Cooperation and Development [OECD], 2019). The campaign for a LW has gained traction due to increasing inequalities associated with a comparatively high cost of living (Arrowsmith et al., 2020).

The importance of public debates regarding contentious issues, such as the LW is reflected in thousands of years of sustained scholarship on rhetoric (the art of persuasion) originating in ancient Mesopotamia, Egypt, Greece, India, and China (Hallo, 2004). Today, the study of rhetoric informs scholarly understandings of how human beings make sense of the world, whilst often seeking to influence the understandings of others (Burke, 1969). A key focus is on the techniques rhetors employ to advise, persuade, and motivate others to think and act in particular ways. Rhetorical techniques involve the use of specific fragments of cultural discourse (e.g., heuristics or tropes) to encourage other people to make quick judgments and accept the plausibility of the rhetor’s position in public debates (Burgchardt, 2010). As such, the rhetorical perspective we adopt is useful in orientating us toward how citizen rhetors participate actively in public deliberations regarding the LW, and present, advocate for, advance, and defend their positions in dialog with opposing views (Burgchardt, 2010). Our study of LW rhetoric focuses on the tropes and devices used by research participants to defend and to persuade others of the merits of one’s own position regarding the LW.

This study is informed by insights from Billig’s (1989) seminal work on rhetorical psychology, which emphasizes the importance of human meaning-making as a communal process that progresses through the reconstruction of conflictual tropes or snippets of discourse. According to this perspective, human beings are storied and argumentative beings who often act as rhetors when making sense of contentious issues through argumentation (Fisher, 1984). This involves rhetors drawing on and contesting available public deliberations as they employ various rhetorical tropes^1^ and devices to influence both their own and other people’s positions on contentious issues. Moreover, by analyzing participant accounts we can explore how rhetors think through contentious issues out loud by combining key points from opposing perspectives (Billig, 1989). Such thinking and arguing involves self-positioning rhetorically within public deliberations by combining and revoicing multiple stock counter arguments (tropes) for and against the LW. This serves to warrant their own evolving positions on the LW in what Billig (1989) refers to as “the spirit of contradiction.” That is, rhetors can often voice what might appear to be contradictory ideas or attitudes as they argue for their own position. This polyvocal orientation to human thought and argumentation guides our exploration of the dynamic complexities of public deliberations regarding the LW, and associated personal, organizational and societal tensions.

This research is also informed by notions of “public deliberation” in which rhetors absorb or concede some counter arguments or tropes as they advance their own positions and interests. The concept of public deliberation is also used to posit that, in democracies, people affected by wage setting decisions (e.g., employers, employees or their representatives) should have a say in decisions impacting upon them (O’Doherty and Stroud, 2019). Ideally, in public deliberations different stakeholders pursue their interests by entering into dialog with those voicing opposing perspectives with a view to reaching collective resolution. As such, public deliberations constitute inter-subjective encounter spaces within which participants may evolve their own arguments to incorporate aspects of counter tropes as they manage complexities and contradictions regarding the LW. As we will demonstrate, interchanges between different perspectives can reach the point where rhetors opposing the LW also re-purpose and voice selective elements of tropes for the LW, and vice-versa. In the process, participating rhetors contribute dialectically to the construction of an organic mini-public (O’Doherty and Stroud, 2019), which is subject to the influence of power relations that shape tensions between employers and employees.

Exploring deliberations within this mini-public is important in addressing these knowledge gaps regarding how the LW is constructed and understood not only by employers, but sector and employee representatives too. This article also responds to a situation in which work is often being touted as the “solution to poverty” (Hodgetts and Stolte, 2017), when most people in poverty are already engaged in work that pays unlivable wages. Further, poor working conditions, including inadequate wages have been recognized as the number one challenge in the world of work [International Labour Organization (ILO), 2019]. This topic warrants our careful consideration.

## Research Context and Approach

Aotearoa/NZ was the world’s first country to endorse a national MW through the *Industrial Conciliation and Arbitration Act of 1894.* The Act established a process of Arbitration to set minimum rates of pay across sectors and occupations as well as requiring compulsory union membership and collective bargaining. This tripartite (employer, union, government) approach lasted until 1991 when the *Employment Contracts Act* deregulated the industrial relations system and contributed to the subsequent collapse of private-sector union representation. This resulted in an imbalance in power relations between employers and workers in wage setting and contributed to increases in poverty.

Legislatively, the legally mandated adult MW introduced with the 1983 Minimum Wage Act was relatively generous and the seventh highest rate in the world in purchasing power parity terms (Schulten and Luebker, 2019). Over time the MW has not kept pace with inflation and its actual value has been eroded. Significant in a recent effort to lift the MW was the election of the Labor-led government in 2017 who announced stepwise annual increases to NZ$20 by 2021, some 27% on the 2017 rate and closing the gap on the LW rate pursued by campaigners. Such increases comprise a response to high living and housing costs that leave many households “…with insufficient income to meet other basic needs” (Perry, 2019, p. 64). In-work poverty rates were between 9 to 12% in 2018 (Plum et al., 2019), and were increasingly recognized as key drivers of negative consequences for employees, their families and communities (Groot et al., 2017; Hodgetts and Stolte, 2017). It is in this context that the LW campaign has gained considerable traction since its formation in 2012 as a means of supporting a basic material standard of living, civic participation and dignified lives for employees, families, and communities (Hurley and Vacas-Soriano, 2018; Carr et al., 2019).

The LW campaign has experienced considerable opposition that often draws from orthodox neoliberal economic “theory,” which is based on equilibrium models of supply and demand, whereby paying above “market clearing” wages is considered uncompetitive and as leading to job losses and/or work intensification (Leonard, 2000). Alternative theories posit that low pay reflects unequal bargaining power, rather than market forces, resulting in a discretionary pay “range of indeterminacy” (Arrowsmith et al., 2003). Concepts such as “efficiency wages” and insights from motivational psychology and social exchange theory also suggest that higher pay can deliver offsetting returns especially in the longer term through better recruitment and retention, returns to training, improved commitment, and employment relations (Card and Krueger, 1995; Searle and McWha-Herman, 2020). Hence, a living wage can benefit both organizations and workers (Carr et al., 2017).

This paper explores these issues by drawing on a major research project running from 2018 to 2021 involving a national quantitative survey of low-paid workers and qualitative interviews and case studies of employers. The employee survey results, presented elsewhere, indicate that wellbeing (both work and non-work) spike at around the living wage figure, suggesting that it could have a significant positive effect. This article draws on 25 semi-structured interviews conducted in late 2018 with sector-level employer representatives, managers at five regional Chambers of Commerce, managers at two city councils (Auckland and Wellington, which have formally adopted the LW), two pay and human resources consultancies, and union representatives involved in the LW campaign. The employer associations represented sectors with a large proportion of low-paid workers (Retail NZ, the Tourism Industry Association, Hospitality NZ, the Food Grocery Council, Federated Farmers, Horticulture NZ, the NZ Aged Care Association, Manufacturing NZ, the Employers and Manufacturers Association) and the peak body Business NZ. The second wave of the research involved four focus groups in December 2020 that again focused on low-wage sectors such as hospitality, cleaning, and retail. Each focus group consisted of senior managers and human resource professionals from non-LW (focus groups one and four) and LW businesses (focus groups two and three).

Qualitative analyses in psychology are often concerned with categorizing the world into lists of mutually exclusive themes. Rhetorical and more impressionistic forms of inquiry also engage in some categorizing, but tropes are not conceptualized as mutually exclusive categories (Burgchardt, 2010; Hodgetts et al., 2021). Rather, tropes are approached as entwined, dynamic and dialogically related common statements or dynamic snippets of shared culture that exist and take shape within rhetorical processes. Likewise, the focus of our inquiry is not on constructing a list of distinct themes. It is on the dialectical interplay of tropes in the contingent formation of rhetorical positions in the LW debate as a dynamic and collective meaning-making process involving the adaptation of these tropes in real time. Although our focus is on interpreting, rather than trying to verify or validate the empirical findings, it is useful for us to offer an account of our iterative process of inquiry that was centered on abductive reasoning or logical inference (Hodgetts et al., 2021).

To initiate this inquiry, the first two authors searched the academic literature and websites featuring arguments for and against the living wage. We then re-read the interview and focus group transcripts independently to explore which tropes featured in participant deliberations. Next, our extensive notes were compared to establish prominent tropes and associated rhetorical devices across the empirical materials and to establish how these were being used in concert by opposing rhetors. We then re-read the research corpus independently and coded extracts of participant accounts relating to each trope. Many extracts were coded in relation to multiple tropes as a reflection of how rhetors combine these when posing their arguments for and against the LW. The first two authors then came together to integrate the preliminary coding and found a high degree of synergy in the exemplars coded against particular tropes. We then explored how participants used combinations of tropes in constructing their positions on the benefits and/or costs of the LW. This open-ended and iterative process that was then extended to additional co-authors who offered critical feedback from their own readings of the participant accounts and deepened the literature base of the article to include, for example, management literature related to this topic. We then met as a team to write through the draft of the article and to deepen the interpretation. A key concern to emerge from this process was how participating rhetors drew on key tropes regarding work and renumeration and combined these dialectically to express stances for and against the LW.

Building on and extending previous thematic work on the LW (e.g., Wood, 1997; Karjanen, 2010; Skilling and Tregidga, 2019), we also considered some of the rhetorical devices employed by participating rhetors in arguing for and against the LW. These include hyperbole (making exaggerated assertions of negative impacts), repetition of fear-invoking emotive assertions (e.g., LW will cause business failures and job cuts), and efforts to construct and foreground contradictions in opposing arguments. Also considered is circumlocution or the use of well-known phrases (tropes) to invoke oversimplified assertions regarding empirically tenuous links between wage rises and job losses. Relatedly, we considered the process of antonomasia, a form of metonymy that is used by participating rhetors to invoke notions of “deserving” and “undeserving” workers and “responsible” and “irresponsible” employers. As we document, antonomasia serves to atomize perspectives on the LW in a manner that detracts from due consideration of issues of public good. Also of key concern was how several rhetors invoked a shared imaginary through references to “the government,” “the market” or “business,” in order to conjure up positive and negative associations for different ideologically orientated groups.

Given the focus on rhetoric and the ways in which participants combined key tropes in crafting their positions in the LW debate, we opted to present our interpretation of their meaning making processes in two sections. The first focuses on arguments for (e.g., fairness, poverty reduction and enhanced organization performance) and the second arguments against (e.g., organizational costs, employee performance and free-market wage setting) the LW. Combined, these sections comprise the core of a new bricolage (Lévi-Strauss, 1966) or exemplified interpretation that is rendered meaningful in the context of the theoretical arguments advanced in the introduction, the interpretative methodological position advanced in this section, and the conclusion that completes this article.

### Arguing for the Living Wage

Key supportive tropes for the LW are linked to fairness, poverty reduction, lower staff turnover, sustainability, and broader benefits to workers, families and society. These were drawn upon throughout the participant accounts by both those taking positions generally for or against the LW. In terms of invoking issues of equity and fairness, the trope of the LW being the “right thing to do’ is prominent in all participants” accounts, whether they support or oppose the LW. In the affirmative, it was often used as a way of introducing complementary tropes, including benefits to the organization, whilst acknowledging contrary concerns. We draw on the arguments of a union representative by way of initial example:

*I guess the drivers are “doing the right thing” for a start and making sure that they’re making an ethical statement about what is an acceptable wage for their staff. And then alongside that comes a whole lot of potential benefits from it in terms of wellness, reduced turnover and absenteeism, loyalty to the organization, those sorts of things*.

Pragmatism is also evident here in that, although a LW might generally be accepted as the right thing to do for employees, it has to be the right strategy for particular organizations. This line of argument was somewhat agreed by participants both for and against the LW, and functioned rhetorically to enable them to present themselves as being reasoned and balanced in their deliberations:

*It’s sort of that space between having a firm, which needs to be profitable, but also having wider goals than just the bottom line.*

Those arguing for the LW acknowledged employer concerns around the financial impacts and issues of profitability. In doing so, these rhetors appropriate concerns from the tropes against the LW around the importance of organizational viability. Proponents of the LW did this by signaling that, although these concerns are important, it is also crucial to consider more than “the bottom line” – rhetorical shorthand for broader societal responsibilities. Also evident is the notion of what this union representative referred to as “good employers” who can gain pride and reputational capital from paying a LW, and contributing to what was termed “a cultural shift” around changing societal expectations regarding fairer renumeration.

Although these quotes come from a union representative, the same accounting practices were provided by many HR consultants and managers, particularly those whose organizations had adopted the LW. In their accounts, the issue of fairness was also contextualized through the use of additional tropes, for example, regarding New Zealand being a low wage economy, despite having a relatively high MW. This rhetorical strategy constituted a less challenging way of promoting the need for a LW by broadening the issue beyond specific organizations to issues of sustainability, prosperity and socio-economic inclusion:

*There’s so many drivers for us as a low wage economy. First of all, there’s the overarching philosophy, which is “it’s the right thing to do.” So, that philosophy propels everything else… It immediately impacts people like care workers… In my view, this is societal change. This is not just the prerogative or the responsibility of employers. It goes far beyond that. It’s embedded across the fabric of society or should be… It has a direct societal impact. (Senior HR consultant)*

Here, references to low wage jobs functions to ground recourse to an abstract philosophical assertion of the “right thing” (read social justice) and the need for changes in the “fabric of society,” which carry material consequences for actual employees. Further, evident in the above extract is the rhetorical positioning of businesses and employers as being embedded in society and, as such, responsible for contributing to the public good.

Pushing this line of argument further, a manager from a power company (focus group two) linked their adoption of the LW to the need to have positive impacts on society as an issue of sustainability (organizational and societal) and “social license”:

*Our sustainability team actually posed this originally, and wedged it into our sustainability framework. It’s very much around care for our people, but also*… *there’s a social license that we have to be able to actually operate.*

Along similar lines, the LW was presented as a mechanism for addressing broader issues of societal equity, with rhetors also emphasizing gender and ethnic pay gaps:

*The one benefit we realized was, that if we bring this* [pay] *up, this is going to benefit more women and is good for pay equity*… *The other thing is the ethnicity equation. We know that some ethnicities are the ones that are in those brackets.* (Council representative)

Exemplified above is how deliberations regarding the LW often extend to the need for societal change to ensure greater equity for different employee groups. Both employee representatives and managers linked pay to addressing inequities, including poverty reduction. As we document in the next section, this is a stance largely contested by sector representatives, suggestive of a “lower common denominator” effect on the part of umbrella organizations in how they respond to or anticipate vociferous concerns that might be raised by some of their actual employer members. What is also signaled here is the pervasiveness of how arguments for and against the LW were entwined and mounted in the context of knowledge of opposing tropes.

For example, in focus group two, a manager for a call center company invoked and challenged arguments against the LW, in general terms and specifically relating to wage compression (differential) effects between new and more established or senior employees. As is acknowledged, these differentials potentially can complicate perceptions of fairness between employees as many would argue that experience and service warrants wage differentials also being the right thing to do:

*The arguments against weren’t relevant anymore. It’s difficult not to pay a living wage when, you know, that effectively is the right thing to do. We were still worried about how some of the long-term staff and team leaders, for example, would cope*… *They’re like a big family and everybody was just thrilled. I haven’t heard any negative feedback from that area at all.*

Here we see an employer raise and then mitigate a key trope relating to wage differentials between newer and long-term staff. This is achieved by first questioning the relevance of arguments against the LW generally and then posing one such argument about wage differentials, and then how they did not receive negative feedback.

An accounts manager in focus group three engaged in a similar rhetorical strategy, but went further to reposition the wage differentials trope as a faulty perception that managers may need to address with their staff:

*We had a bit of a talk in the beginning that people were like, “Well I’ve been here years, I’m gonna be getting like a few dollars more* [than new staff]. *It just doesn’t feel right.” You go like, “Well stop comparing! You were happy like a month ago when you were getting paid that wage. You’ll be happy next month when you’re paid that wage.” And why can’t everybody have a good wage? Just because you’re earning this much more than the other person, don’t keep looking at it like that. Look at it like everybody’s earning this much and then everyone can go off and go home and garden, or dance or do whatever they want to do.* [We are] *trying to change the mindset.*

Combined, the rhetorical strategies of these business leaders (particularly the second) offer a glimpse into how tropes regarding fairness and wage differentials can be reworked to support the LW and its impacts on different staff groups. The LW is also presented in the second case as a key mechanism for this self-perceived responsible organization to achieve its humane goal of all employees being able to earn enough to enjoy quality of life. Relatedly, many employers also differentiated the living from the minimum wage, indicating that moving to the LW constituted evidence for a responsible employer:

*It’s almost embarrassing for an organization to turn around and say, “We pay minimum wage.” It’s kind of like, “Oh, really?” I think especially when, you know, the difference in terms of the impact on somebody’s life between paying minimum wage and paying living wage. It’s really difficult to argue out of that. And I get there’s mum and dad businesses out there that are struggling, but really it’s almost a onetime cost if you forecast it into your business. (Focus group two)*

Central to such arguments is the difference between the MW and LW, and how increasing pay to a LW is constructed as a responsible move that can enhance an organization’s reputation (cf. Haar et al., 2018). It is also interwoven with the assertion that organizations can benefit from meeting changing public expectations around pay and employer responsibilities.

Issues of fairness for employees were also extended to benefits for good employers, including improved relationships with staff and reduced turnover. In this line of argument, the LW was often presented as a sensible or logical strategy in that it provided a win-win situation for employees and employers. In the service sector, it was asserted that these benefits also extended to customer and community relationships as well as potentially positive labor market and employment effects:

*I notice that there’s a bit of a shift now to operators paying a bit more for both attraction and retention. That’s probably one of the drivers*… *It helps to improve their standing within the local community*… *Again, both those things are perhaps indicative that employers are starting to see the role that LW or fair wage plays in both retention and attraction, but also working with their local host communities.* (Sector representative)

This extract also reflects the importance of local embeddedness and organizational reputation in the tourism industry. Significant too is the linking of a LW to a “fair wage,” again reflecting the currency of the “right thing” trope in terms of benefits for employees, organizations, communities and society.

This perspective was echoed by a brewery manager in focus group three who connected the LW to the brand, organizational values and broader issues of social responsibility:

*As a brand and in terms of the values it stands for, from the outset has always been very community minded*… *That’s what predominately drove us to seek accreditation with the LW*… *We wanted them to have a sustainable lifestyle, and*… *a duty to care for them*… *It received a huge positive response from our team internally and externally*…

Another manager of a security firm (focus group three) argued that his organization was proud to pay staff a LW because they deserved it, and this also returned retention, reputational and growth benefits to the organization (*cf*. Haar et al., 2018). After proposing that introducing the LW reduced their staff turnover from 100 to 20% per year, this rhetor proposed:

*That alone makes a huge impact to our business. It costs us 5,000 to 10,000 dollars to train a patrolman*… *We can feel a little bit better knowing we’re giving some of that money back to the people that really deserve it*… *We have a lot of organic growth in the business and it is word of mouth. What people are beginning to see out there is if they pay for a quality product then they’re gonna get it*… *It’s testament to having skilled staff, experienced staff that stay with you, because they’re paid well and they’re looked after.*

Such accounts of employee deservedness, worth, retention and business growth remind us that organizations operate in ecosystems where reputation for equity, social responsibility, and skill competence can generate mutually beneficial and sustainable returns, or what the ILO Decent Work Agenda and the SDGs term “shared prosperity” (United Nations, 2021, SDG-8).

The issue of business sustainability was often used to argue both for and against a LW. In the former, anticipating concerns around financial sustainability, rhetors proposed that well-run businesses are more likely to be able to afford the LW. This rhetorical reframing placed primary responsibility on employers to run their organizations well so that they could afford to do the right thing. This stance is in direct opposition to the common trope against the LW that organizations will become unsustainable if they try and introduce the LW. Again, some hedging work is used in such proposition in terms of acknowledging the need for some firms to increase revenue from customers, which invokes the consumer as a social actor who can contribute to the LW by being willing to bear increased costs for products and services:

…*Successful businesses have to be resilient enough to be able to cope with wage increases over time, provided they can offset that cost to the consumer in some way*… *The consumer has the opportunity to increase its behavior, then businesses should be structured well enough to be able to increase their costs as well.* (Focus group three)

Evident in the accounts of several such rhetors was the need for organizations to be managed well enough to be able to afford a LW, placing the responsibility regarding issues of affordability to employers who design work systems (cf. Levin-Waldman, 2000). Subversion of counter arguments for the need for productivity increases to pay for the LW was also evident in the contribution of a manager from a hospitality firm in focus group three:

*And why did they [the business] do it?. Definitely not anything to do with productivity or squeezing the most out of their workers. You know, people’s lives are important and your job is just your job*… *You’ve all got your own lives and they just wanna encourage people to be able to live them*…

In this exemplar, the rhetor creates a rhetorical opposition between drivers for the LW being productivity gains and needing to care for one’s staff by not necessarily intensifying their workloads. Also evident is the relativizing of a job in terms of recognizing the importance of broader concerns such as workers being able to live quality lives.

In sum, this section has documented the complimentary use of various tropes in support of the LW as a means of benefiting employers, employees and broader society by addressing issues of equity and economic inclusion. Evident is how arguments for the LW are also crafted to subvert dominant tropes against the LW. This involves rhetors presenting themselves as reasonable and pragmatic by acknowledging and responding to the concerns of “some” employers regarding financial and operational matters, and in voicing the need to ensure the LW is the right strategy for particular organizations. Additionally, pro-LW rhetors also expand the debate out beyond specific operational concerns for employers. Participants promote the importance of considering the broader societal responsibilities that “good employers” accept. This serves to position “good” and “bad” employers differently in relation to the public good and changing societal expectations regarding the need for socio-economic inclusion and poverty reduction.

### Arguing Against the Living Wage

Key tropes against the LW related to business affordability, job losses, unrealistic employee expectations in life, undeserving employees, and the complexities that LW may impose on businesses (pay relativities). LW opponents also argue that there are better alternatives for addressing poverty (reduce housing costs), the importance of individual choice and fairness, free market autonomy, and that morality has not place in business decision making. Prominent tropes relating to negative impacts on jobs and business failures were invoked through the rhetorical technique of hyperbole and presented as factual “common sense,” despite a lack of empirical support (Karjanen, 2010). Another key rhetorical technique was the use of circumlocution to oversimplify links between wage rises and job losses, and to dismiss or ignore counter evidence. Implicit to rhetoric against the LW is a shift in responsibility from the employer to the employee who must become more productive to ensure the business is able to pay a LW. Overall, the strategic use of key tropes and rhetorical techniques served to marshal uncertainty regarding the benefits of a LW, cultivate fear regarding possible job losses, and to bring into question who is responsible to resolve in-work poverty.

Rhetors who oppose, or are more cautious around the LW, were less likely to be actual employers and more likely to be sector representatives or from chambers of commerce. In arguing against the LW, these rhetors demonstrated awareness of tropes supporting the LW considered above, which they repurposed to align with their own oppositional positions. Their undermining of the LW was often both overtly affronting as well as more subtle, as is the case in the following extract:

*I think the benefits are a better-engaged workforce, potentially. Although there’s no guarantee of that either.* (Chamber of Commerce)

Such assertions reflect how many opposed rhetors did not necessarily contest key arguments in support of the LW, but rather subtly minimalized benefits as only possibilities. In so doing, they brought these benefits into question. Such approaches also extend to subtle challenges to notions of fairness and the LW as a means of addressing poverty, often with reference to pay differentials and organizational viability:

*The ultimate goal is for people to get a fair wage for a fair day’s work. It’s also about trying to reduce that poverty gap. The key to some of that is, are we just squashing the lower and middle band closer and closer together, because let’s say your wage goes up, and then each level has to go up. So, if your office worker now goes up by NZ$5, that means your middle manager has to go up by NZ$5*… *Yes, the pay increases are absolutely positive for the staff themselves, but the implications that it has for providers’ viability is quite significant.* (Chamber of Commerce)

Through such extracts, we see a general acknowledgment of acceptance of the LW in principle as a possible means of addressing in-work poverty. However, the challenge to the LW comes in the form of rhetors strategically raising various practicalities and complications for organizations that need to be worked through before a LW can be introduced sustainably. Further, whilst the need to address in-work poverty is accepted, such rhetors support the *status quo* by offering no convincing alternatives for addressing in-work poverty and focusing on possible complications in terms of pay differentials.

As we will show below, this line of argument often then morphs into assertions regarding organizational viability, job losses, issues of employee value or productivity, and the targeting of the LW to employees who are deemed “deserving” by employers. For example, a senior HR consultant argued against the LW through fear regarding job losses and business failures, and to prompt the need for productivity gains:

*My concern is for the bottom end of the market, the companies that struggled to pay that. They will go out of business. One employer I’m particularly thinking of…, he’s already said to me, “I won’t employ as many people.” It’s easy. “To keep my cost of labor down I’m not taking the risk on these idiots. I’m going to employ less people, so the ones that I’ve got are going to have to work harder.” This particular employer has got his business up for sale at the moment. If it doesn’t sell, he’s going to close it down because he’s sick of the HR issues and how hard it is to get hard-working employees.*

The ethics of referring to employees as “idiots” by proxy aside, such extracts were at times quite threatening in tone and reliant on reprisal. This rhetor presented the case of an undisclosed business as a rhetorical means of supporting a truth claim that businesses are worried, while some employers are looking to sell or wind up their businesses because of perceived existential threats from rising employment costs. This line of argument comprises a more refined variation of the “it will cost jobs and businesses” trope. Interestingly, it was not advanced in the accounts from organizations that had adopted the LW.

Arguments against the LW are sometimes informed by stereotypes of undeserving, dysfunctional, unskilled, and more commonly unproductive workers. Not paying a LW was often recast as equitable due to undeserving workers not being productive enough:

Productivity is what the whole economy needs… It’s very poor. Employees taking drugs and living from week-to-week and drinking their pay… and not turning up on the weekend or on Monday. All those issues need to be addressed to improve productivity. Last time I checked 20 percent of the New Zealand population was illiterate or partially illiterate. (HR consultant)

This line of argument is often extended through the proposition that at best the LW should be a targeted intervention to employees who are more work orientated, motivated and productive so as to avoid the type of undeserving workers invoked above from gaining a “free ride” (Karjanen, 2010). This “undeserving employee” trope was also contrived in more subtle terms by other rhetors as “*let’s pay people for the contributions that they make*” (Retail manager, focus group four). Individualizing issues of pay with reference to employee deficiencies distracts attention from issues of employer social responsibility and social license (Levin-Waldman, 2000). Further, national deficiencies in productivity can be presented as the result of undeserving employees transgressing traditional norms around hard work and sobriety, rather than insufficient wages and poor work conditions (Carr et al., 2011; Blumkin and Dansiger, 2018). Such rhetoric also positions “undeserving” workers outside the scope of justice (Hodgetts et al., 2020) because they have not met the moral responsibilities of productive workers. Positioning a group outside the scope of justice means that they can be denied the same rights and privileges as people located within the scope.

Our attention is drawn here to the rhetorical strategy of antonomasia that involves the use of a form of metonymy or key words that stand in for an actual object. In this context, the classic distinction between the “deserving” and “undeserving” poor is used to contest the fairness of a LW and to shift responsibility for poor pay onto employees (cf. Skilling and Tregidga, 2019). On the surface it may well be reasonable that if employees want increased pay then they need to increase their skills and work harder. However, this line of argument is also used to warrant the *status quo* that features unlivable wages and exploitative employment practices whereby employees have to work harder with no guarantees of increased pay. Such accounts serve to naturalize unlivable wages as meritocratic responses to individual performance and omit the fact that many “deserving” workers are also currently paid below the LW.

Such rhetorical sleights-of-hand were used by several rhetors opposed to the LW to repurpose notions of fairness and to argue against the LW on the basis of performance. Central to such rhetorical repositioning are tropes relating to productivity, affordability and possible negative consequences for businesses. While not wishing to totally dismiss employer concerns underlying these tropes, it is important that we consider issues around increases in labor and associated costs that are carried by organizations, and which were associated by several rhetors with issues of sustainability, profitability, and job losses:

*They [businesses] restructure their approach to cost, which … can be laying off people, it can be in fact introducing more technology to offset the labor components, and a whole bunch of cost-based responses. (Sector representative)*

Such extracts reflect an assumed cause and negative effect pathway from the introduction of a LW and job losses and automation. They also carry a false equivalency in threatening that a LW “logically” equates to such negative outcomes:

Unemployment will go up because those businesses will close. There are consequences – cause and effect … If it’s regulated, it [LW] will force companies to close in the hundreds or thousands in New Zealand. That means unemployment is going to go up and the cost of inflation will rise. (Sector representative)

This negative cause and effects trope was widely presented as common sense. Omitted from such assertions was how many businesses could absorb increased wage costs by reducing profits to shareholders, reducing salaries and bonuses for higher earning staff, as well as through more benign process efficiencies (Kenway, 2016; De Bievre, 2018).

This is not to say that rhetors opposed to the LW did not acknowledge organizations that had adopted a LW without job losses. These rhetors raised important queries around complexities in cost structures across different organizations and how some sectors and organizations may be better placed to adopt the LW than others. However, they also worked to rhetorically mollify the positive experiences of LW organizations by associating the LW with monopoly or public organizations, which faced less competition and could pass on added costs:

*Those who have been advocates for a living wage have tended to be organizations like councils and agencies that aren’t responsible for the generation of their own incomes.* (Chamber of Commerce)

Such extracts reflect rhetorical efforts to undermine the credibility of the messenger (public sector organizations), and in doing so dismiss evidence for the benefits of the LW. Also omitted here are the cost restraints faced by such organizations in terms of how much they can actually raise their incomes through increased taxes and other such means.

The efficacy of a LW was also brought into question through recourse to prominent societal tropes regarding the high cost of living. Many argued that a LW will not address in-work poverty because of the rapidly rising cost of housing, and legitimately proposed that the government should look at alternative strategies, such as addressing rental costs. The issue of income and cost of living is then diverted from one of income to one of failed public policy:

*The reason is of course, that New Zealand has, I think, probably the highest MW in the world*…, *not just nominally, but if you normalize it for currency. The reason why that’s not enough is not because the figure is too low, it’s you can’t afford to live here on it; and the reason why you can’t afford to live on it is accommodation costs are too high. So, on the one hand, for unions and the government, it’s very convenient for them to frame this as the miserable employers are not paying enough; when, in fact, the social policy and the public policy issue sits really around other things.* (Sector representative)

The same rhetor went further to associate the cost of living to unrealistic employee expectations and personal differences in what constitutes a dignified quality of life. In doing so, they re-imagined a past where employees were more “realistic” about what they actually needed:

*What is a good life? There was a time if you were dry and warm and not too hungry you were okay, because the other guy probably was in bare feet out on the street. Today, that’s not good enough; we expect to have access to broadband and a whole lot of things that we think are absolutely the minimum requirement to be living… The problem with the living wage is, who’s living? It’s a very imprecise measure and it doesn’t actually tell the whole story, because my life and your life will be different. I might want to live in a cave somewhere and that living wage is going to look like a king, but if I’ve got three kids, and I’ve got a sick mother-in-law at home or something, that’s not nearly enough to actually live with dignity. (Sector representative)*

The “people’s needs differ” trope often manifests via a selective hypothetical comparison between personal expectations, which serves to sidestep issues of material hardship that come with unlivable wages and a high cost of living. The focus is shifted from the adequacy of income to “unrealistic” employee expectations. This line of argument is foundational to efforts to relativize the LW as an issue of personal lifestyle, rather than equity.

Many rhetors, likewise, posed legitimate queries and objections regarding how the LW is calculated. However, these also functioned rhetorically to shift the focus toward technicalities and the perceived biases of LW proponents. This enables rhetors opposed to the LW to avoid engaging with the broader issues of equity, fairness and exploitation (Karjanen, 2010). Such strategies were also implicated in notions of the sanctity of free market autonomy (Skilling and Tregidga, 2019), to the extent that it was repeatedly asserted that the Government setting a LW is inappropriate when, in fact, such wages are not state mandated. What is evident in such assertions is the use of arguments against the increasing MW, which is set by government with the voluntary LW, which is not:

*This particular government is trying to artificially increase wages without having any of the other factors in place first, or a plan for any of those other factors … I mean, maybe we’ll be fine and maybe we won’t. I don’t know. But, I think that’s an unbelievable risk to take when everything that you read says that’s not the way that it should be done. (Sector representative)*

Several rhetors appealed to popular imagery of “the Government,” along with unions disrupting what are asserted to be the “natural” market self-regulation of wages. This trope has been used against the MW in the United States (Wood, 1997) and is used in Aotearoa/NZ to assert that LW proponents are socialist Christians who are biased and should not be emboldened to interfere in free market wage setting:

*I can’t understand why somebody hasn’t formally come out with another version of the LW that is not so reflective of a faith-based research organization and a trade union who have their obvious biases. (HR consultant)*

Asserting that opponents are biased infers that one’s own position is somehow more objective. We also discerned the rhetorical strategy of oversimplifying the world into dichotomies between businesspeople and Christians. The latter are positioned as existential threats to the market, business and economy, and ultimately the welfare of employees.

In terms of the sanctity of the free market trope, it is important to note that many employers did not fully embrace this line of argument. For example, reflecting arguments for the LW explored above, a senior manager from focus group three proposed that “the market” is actually shifting toward the expectation of a LW, at least in part, due to shortages of skilled migrant labor (exacerbated by the pandemic) as well as broader benchmarking away from the MW:

*In terms of recruitment, which I do a lot of, I think the market was starting to expect a living wage from employers. I think particularly the kind of brand that we like to aim for, a lot of talent we were recruiting were already demanding that.*

In the extract above the market trope is undermined somewhat through the assertion that the market and society are actually shifting toward expecting a LW. This reworking of a key trope against the LW also undermines the artificial division between the market and society, which is used by other rhetors to privilege self-interest over collective interest, and to ignore how indebted businesses actually are to the societal infostructure upon which they rely to operate. It also remines of the importance of nuance and diversity in employer perspectives.

Above, we have presented arguments against the LW that respond to opposing views, and in particular dispute the meaning of fairness, affordability of wage rises, and foreground potential negative consequences for employers and employees. These rhetors also shift the focus from the LW to alternative strategies for addressing poverty and emphasize the importance of personal choice and the sovereignty of the market over perceived government interference in wage setting. Whilst questioning what is presented as socialist and moral arguments for the LW, several rhetors voice conservative moral distinctions between the deserving and undeserving poor, which are used to question justifications for the LW based on concerns regarding unrealistic expectations, low productivity and work ethics among the underserving. This rhetoric work functions to differentiate employees to promote uncertainty regarding arguments of equity that support the LW. It also shifts responsibility away from employers and onto employees who need to become more productive and realistic. The appropriateness of the LW as a form of poverty reduction is questioned through the introduction of fear regarding negative consequences, such as job losses, and the questioning of why employers are being targeted to reduce poverty and not groups such as landlords who often increase the cost of living.

## Conclusion

Little is known empirically about the rhetorical relationship between employer and employee stakeholder understandings of the living wage, particularly with regard to low pay sectors (Werner and Lim, 2016). This article reveals considerable overlap between positions for and against the LW that are worked through rhetorically within the dynamic accounts of participating rhetors. For example, a dominant trope across opposing rhetors was that the LW may well be the right thing to do to support a dignified life for employees. Relatedly, both sides acknowledge that not all employers may have the means to pay a LW. Where rhetorical positionings tend to differ is in terms of affordability, what consequences a LW has, and how it can be funded. Understanding such rhetorical complexities is crucial for engaging with issues of equity, fairness and business social responsibilities in wage-setting deliberations and work-based poverty reduction efforts.

This article also documents the influence rhetoric can have in stakeholder contributions to public deliberations regarding the LW. These accounts are also shaped within broader socio-political contexts and power relations that are reproduced, supported, justified, and challenged within these deliberations. Correspondingly, Bitzer (1968) as argued that rhetoric is central to the construction of reality through the recreation of key aspects of public discourse (deliberation) and the perspectives and actions of those involved. By documenting rhetoric within public deliberations regarding the LW, we are able to open up and examine the multiple and competing realities on offer (Burgchardt, 2010). To this end, we have documented how various tropes are employed by key stakeholders (rhetors) to influence and explore the plausibility of and adopt similar positions regarding the need for and consequences of the LW (O’Doherty and Stroud, 2019). This is important because public deliberations can have actual material impacts in terms of whether or not the LW is adopted by particular organizations, and what consequences this has for organizations and employees, their families and civil society. A rhetorical perspective also orientates us toward the relationship between key snippets (tropes) of these deliberations and to avoid presenting these as a list of mutually exclusive categories of argumentation. We have demonstrated how different tropes become entangled in dynamic and emergent ways within competing positions in public deliberations regarding the LW.

Beyond documenting key tropes in public deliberations regarding the LW, a key contribution of this article is documenting how these are employed rhetorically. This is important because human meaning-making is often embroiled within rhetorical practices that are not always conducted in accordance with the dichotomous logic of either or, which is often employed in psychology to demonstrate how people all make sense of the world. In actuality people often entwine existing tropes from public deliberations in agentive and novel ways to extend their understandings of arguments regarding the LW (Billig, 1989). Supporting the importance of this focus, several of the tropes we have explored are also evident within previous research on public documents in the United States, United Kingdom and NZ (Wood, 1997; Levin-Waldman, 2000; Karjanen, 2010; Skilling and Tregidga, 2019). These include the sanctity of the “free market” in wage-setting, pay rises costing jobs, and concerns regarding productivity. Rhetors on both sides have heard, thought about, and draw on opposing arguments to promote their own agendas rhetorically.

A prominent example was how opponents of the LW propose that increasing wages can be a positive phenomenon, but qualify this ascription to some deserving workers, arguing that it can also cost others their jobs. Although there is little empirical evidence of resulting job losses attached to significant increases in low pay (Jardim et al., 2018; Aitken et al., 2019) and emerging evidence to the contrary (Parker et al., 2015; Kenway, 2016), this is omitted in this combination of tropes. Further, the persistence of tropes such as “the LW will cost jobs” demonstrates the rhetorical strategy of circumlocution (use of a few well-known phrases to narrow engagements with this complex issue). It also exemplifies how current public deliberations are populated by misinformation, half-truths and threats of business closures if the LW is normalized. Correspondingly, one might expect that many employers who want to “do the right thing” by their employee’s express uncertainties about the potential consequences of such a move (Karjanen, 2010). What also becomes apparent is how current public deliberations regarding the LW do not meet expectations for reaching consensus or a fair resolution through open dialog (O’Doherty and Stroud, 2019). Although rhetors may have heard counter arguments, they may not necessarily be listening to these or be as willing to shift their own positions. Contestation prevails.

Wood (1997) also proposed that central to public debates about renumeration are issues around the adequacy of the free market in wage settings. Little evidence supports the assertion that markets are naturally egalitarian social phenomena, free from power differential-based distortions or the best mechanism for wage setting (Karjanen, 2010). The MW is not currently a LW, partly due to the contemporary asymmetrical power relations between employers and unions that have artificially deflated wages over the past few decades of neoliberalism (Arrowsmith et al., 2020). Unlivable wages and in-work poverty can also be read as a failure of the market to set an equitable wage floor. Further, it can be asserted that low-wage employers going out of business constitutes a valuable form of market correction.

Previous research into the LW debates in the United States and United Kingdom (Karjanen, 2010) found that those opposed tended to work harder to divert the debate from issues of fair pay and social responsibility in addressing in-work poverty. In considering key rhetorical practices in Aotearoa/NZ, we can see how issues of fair pay and poverty reduction were not avoided, but rather were presented in the context of other important issues of fairness, including pay differentials and the need to address the high costs of living. These issues are presented as beyond employer control, and therefore responsibility. As such, rhetors against the LW do not always directly challenge the equity or fairness trope. Rather, they employ rhetorical strategies and devices to appropriate, circumvent or decontextualize such affirmative tropes. In doing so, they can concede that in-work poverty is a negative phenomenon, the solutions to which lie elsewhere in society. There are some grounds for this position, which reworks cost of living concerns shared by many LW campaigners. Our analysis reveals that concerns regarding pay and the cost of living are not mutually exclusive. In policy terms, it is necessary to raise wages where this can be done sustainably, but also to prevent these gains to employees from being syphoned off by landlords, for example.

Whilst also hearing the concerns of opposing employers regarding issues of productivity and the needs of small enterprises, LW proponents raised broader considerations regarding organizations’ social responsibilities that rhetorically re-position business as accountable to society. These rhetors then propose that there are growing expectations that employers should pay employees enough to live with dignity. In doing so, they do not deny counter arguments that wage increases may have challenging implications for employers. Rather, they present the LW as a key mechanism toward a fairer or more equitable society. Given such rhetorical efforts in current public deliberations we are heartened that both sides are familiar with opposing perspectives.

## Data Availability Statement

The datasets presented in this article are not readily available because the qualitative data collection is not intended to be shared due to sensitive information. Requests to access the datasets should be directed to DH, d.j.hodgetts@massey.ac.nz.

## Ethics Statement

The studies involving human participants were reviewed and approved by Human Ethics Committee Northern, Massey University. The participants provided their written informed consent to participate in this study.

## Author Contributions

All authors contributed to manuscript revision, read, and approved the submitted version.

## Conflict of Interest

The authors declare that the research was conducted in the absence of any commercial or financial relationships that could be construed as a potential conflict of interest.

## Publisher’s Note

All claims expressed in this article are solely those of the authors and do not necessarily represent those of their affiliated organizations, or those of the publisher, the editors and the reviewers. Any product that may be evaluated in this article, or claim that may be made by its manufacturer, is not guaranteed or endorsed by the publisher.

## Footnotes

1 For our purpose, tropes comprise figurative, metaphorical, recurrent and readily recognizable shorthand figures of speech, narrative plot elements or points of argument that populate ongoing debates (Sandberg, 2016).
